# Self-assembled Bismuth Selenide (Bi_2_Se_3_) quantum dots grown by molecular beam epitaxy

**DOI:** 10.1038/s41598-019-39821-y

**Published:** 2019-03-04

**Authors:** Marcel S. Claro, Ido Levy, Abhinandan Gangopadhyay, David J. Smith, Maria C. Tamargo

**Affiliations:** 1 Department of Chemistry, The City College of New York, New York, NY 10031 USA; 2 Ph.D. Program in Chemistry, The Graduate Center of the City University of New York, New York, NY 10016 USA; 3 School for Engineering of Matter, Transport and Energy, Arizona State University, Tempe, AZ 85287 USA; 4 Department of Physics, Arizona State University, Tempe, AZ 85287 USA; 5 INL – International Iberian Nanotechnology Laboratory, 4715-330 Braga, Portugal

## Abstract

We report the growth of self-assembled Bi_2_Se_3_ quantum dots (QDs) by molecular beam epitaxy on GaAs substrates using the droplet epitaxy technique. The QD formation occurs after anneal of Bismuth droplets under Selenium flux. Characterization by atomic force microscopy, scanning electron microscopy, X-ray diffraction, high-resolution transmission electron microscopy and X-ray reflectance spectroscopy is presented. Raman spectra confirm the QD quality. The quantum dots are crystalline, with hexagonal shape, and have average dimensions of 12-nm height (12 quintuple layers) and 46-nm width, and a density of 8.5 × 10^9^ cm^−2^. This droplet growth technique provides a means to produce topological insulator QDs in a reproducible and controllable way, providing convenient access to a promising quantum material with singular spin properties.

## Introduction

The electronic confinement in all three dimensions of semiconductor quantum dots (QDs) leads to unique quantum properties. A discrete energy spectrum is produced, and the confinement impacts how the electrons interact with each other and to external influences, such as electric and magnetic fields. These quantum effects can be tuned by changes in the sizes of the dots or the strength of the confining potential. Such QDs are used in many diverse applications, spanning from devices such as lasers^1^, solar cells^2^ and photodetectors^3^, to the study of new physical phenomena, such as single photon interactions^4^ and spin manipulation^5^.

In the well-known common semiconductors, QDs are typically created using heterostructures defined by lithography or by self-assembled crystal growth. QD growths by molecular beam epitaxy (MBE) in Stranski-Krastanov mode^6^ and by droplet epitaxy^7,8^ are the most commonly used techniques for Si-Ge, and III-V semiconductors. QDs could also be formed by the electrostatic confinement of 2D electron gases^9^. However, in materials where the electronic states are protected by time-reversal symmetry, i.e., Graphene^10^, and the class of materials known as Topological Insulators (TI), the electrostatic potential cannot confine or scatter electrons as usual, a property known as the Klein paradox^11^. Thus, quantum confinement in these materials can normally only be achieved by the formation of 0D nanostructures.

Three-dimensional TIs, such as Bi_2_Se_3_ and related materials, are insulators in the bulk form, usually with a narrow band gap. However, they have surface states with spins that are locked with momentum and protected by time-reversal symmetry^12^. Thin films possess favorable bulk to surface volume ratio to enhance the TI properties and have applications that include quantum computing, dissipation-less electronics, spintronics, enhanced thermoelectric effects and high performance flexible photonic devices^12^. Among the many TIs, Bi_2_Se_3_ is particularly interesting because its band gap is larger than those of most other TIs, and the experimentally verified Dirac cone is at the gamma point^13^. These materials exhibit a tetradymite crystal structure with Se-Bi-Se-Bi-Se units, commonly referred to as quintuple layers, that are bonded together by van der Waals forces^14^. Bi_2_Se_3_ can be synthetized as bulk crystals, thin films or nanoparticles by several methods^15^. Bulk crystals have been synthetized by Bridgman–Stockbarger^16^, flux^17^ and other methods, and then chemically or mechanically exfoliated to study their properties as thin films^18^. Excellent results have been achieved this way. Unfortunately, these methods have poor controllability and low yield, and cannot be applied for large-scale production or large-area applications. Solution-based processes have also been applied successfully to synthesize nanoparticles and to study their novel optical properties, from photothermic absorption^19^ to nonlinear optical properties such as saturable absorbers, which have applications in photonics^20^. The chemical vapor deposition (CVD) method can produce relatively high quality nanomaterials on a large scale^15^. However, none of these methods can achieve the purity and controllability of MBE^14^. In MBE the material grows in ultra-high vacuum using only fluxes of high purity Bi and Se. High quality Bi_2_Se_3_ has been grown successfully by MBE on different substrates^21^ and, due to the ability to controllably dope materials achieved with MBE, the Quantum Anomalous Hall effect could be observed^22^. Additionally, MBE-grown QDs can be readily incorporated into multilayers and heterostructures with other TIs^23^ as well as with 3D materials^24^, thus greatly expanding the range of possibilities for novel physical phenomena and device applications.

As with other materials, we expect that some properties of TIs and Bi_2_Se_3_, especially those related to spintronic and quantum computing, can be enhanced by quantum dot confinement^25–27^. In lithographically defined QDs, the quantum confinement was already previously demonstrated^28^. However, to our knowledge, very few experiments have been done to investigate the effect of the quantum confinement of TIs. The lack of adequate means to fabricate mesoscopic structures in a reproducible, high purity and controlled way is identified as a major obstacle. Nonetheless, the MBE growth of Bi_2_Se_3_ occurs by van der Waals epitaxy^29^ since it is a layered material, and strain cannot be used to induce QD formation by the Stranski-Krastanov mode as in other materials. In this work we demonstrate a viable method to create self-assembled quantum dots of Bi_2_Se_3_ by molecular beam epitaxy based on the droplet epitaxy technique.

## Results and Discussion

The samples were grown in a dual-chamber Riber 2300P system equipped with *in situ* reflection high-energy electron diffraction (RHEED). The GaAs semi-insulating (001) substrates were prepared by oxide desorption in the first chamber, followed by the growth of 200 nm of GaAs at 575 °C by conventional MBE. The substrates were cooled under an overpressure of Arsenic and a flat c(4 × 4) surface reconstruction, typical of As-terminated GaAs, was observed by RHEED at the end of this process. The substrates were then moved under ultra-high vacuum (UHV) to the second chamber for growth of the Bi droplets and the QDs. The background pressure of the second chamber, where the droplet and QD growth occurred, was 1–4 × 10^−10^ Torr during growth. High-purity 6N bismuth (Bi) and selenium (Se) were provided by a RIBER double-zone cell for Bi and a Riber VCOR cracker cell for Se. Fluxes were measured by an ion gauge placed in the path of the fluxes. The estimated flux ratio was 10:1 Se to Bi. The temperature used for the Bi source was 730 °C and the beam equivalent flux (BEP) was 2 × 10^−8^ Torr. These conditions led to an average growth rate of 32 nm/h for Bi_2_Se_3_ by van der Waals epitaxy.

**Figure 1:**
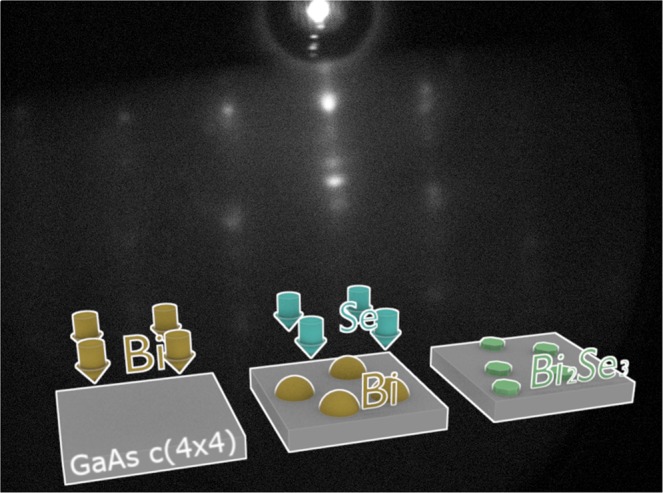
Spotty (1 × 1) RHEED pattern observed after formation of Bi_2_Se_3_ QDs. The inset illustrates the steps involved in the growth sequence.

The growth of the bismuth droplets was initiated by opening the Bi shutter when the prepared substrate temperature reached 250 °C, and the flat c(4 × 4) surface reconstruction was still visible. During the first few minutes, a circular ring started to appear in the RHEED pattern and the streaky pattern from GaAs began to fade, indicating the formation of amorphous material on the surface. The RHEED pattern became totally diffuse after 15 min with barely visible spots that appeared in some orientations. For the droplet sample, the Bi shutter was closed at that time and the sample was cooled down and extracted for analysis. For QD formation, after the Bi shutter closure, the Se shutter was opened and the sample was exposed to Se flux at the same temperature of 250 °C. The RHEED pattern changed from the diffuse pattern to a “spotty” (1 × 1) reconstruction pattern. After approx. 2 mins, the RHEED pattern was totally transformed into the pattern shown in Fig. 1. The droplets were exposed to the Se flux for 40 min in total to ensure the complete formation of the Bi_2_Se_3_ and to minimize Se vacancies. However, since the RHEED was stable after the first few minutes of growth, it should be possible to reduce this time considerably in future studies. The inset in Fig. 1 illustrates the steps that occur during the QD growth.

**Figure 2:**
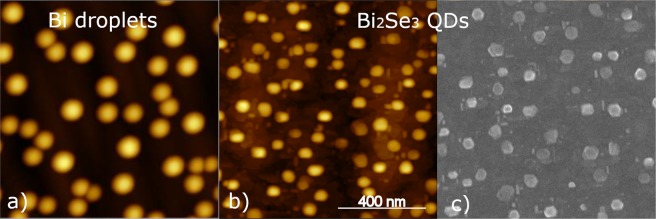
AFM images: (**a**) bismuth droplets; (**b**) Bi_2_Se_3_ QDs; (**c**) SEM image of Bi_2_Se_3_ QDs. All pictures have the same scale.

Figure 2a shows an atomic force microscope (AFM) image of the Bi droplets. They have semi-spherical shape, with average height of 29 nm and density of 4 × 10^9^ cm^−2^. Despite differences in the growth conditions, these results are comparable with Bi droplet growth reported in the literature^30^.

From the AFM image, (Fig. 2b), the QDs are measured to have an average height of 12 nm (12 quintuple-layers) and an average diameter of 46 nm, and the QD density is ~8.5 × 10^9^ cm^−2^. The size achieved is sufficient to preserve the characteristics of 3D TIs while, at the same time, some confinement is expected according to theoretical predictions^25^ and previous measurements^28^. The transition to TI in thin films occurs at a thickness greater than about 6 quintuple layers^31^. In comparison to the radius, height, density, and finally the total volume of the Bi droplets, the QDs are smaller, shorter and of higher density. Moreover, the QD density is approximately twice the droplet density. Several factors could explain this difference, such as the presence of some Bi desorption during the annealing process, ripening of droplets during QD formation, and changes in the Bi droplet size due to surface migration of Bi during cooling. These effects were observed in self-organized growth of nanostructures in other materials^32^. Due to convolution of the probe tip and the QDs, the QD shape is blurred in AFM images. However, from SEM images, as shown in Fig. 2c, the facets and the hexagonal shapes of the QDs are still preserved. Notably, AFM images repeated after samples were stored at room temperature, in ambient atmosphere, for over nine months, were unchanged from the original ones taken immediately after growth, indicating that the QDs are stable. Based on previous results for Bi-droplet formation on GaAs surfaces^30^, it is anticipated that the QD size can be varied in a controlled and reproducible manner by adjusting the growth parameters, such as Bi deposition temperature and time. We also note that due to the absence of strain as the driving mechanism for QD formation^33^, defects such as stacking faults, which appear in Stranski-Krastanov QDs, are not expected in QDs made by the droplet epitaxy technique, making this approach highly desirable.

To establish the QD stoichiometry, the composition at the surface was measured using energy dispersive x-ray (EDX) analysis coupled to an SEM. Despite strong substrate signals from Ga and As, values of 2.3% of Bi and 3.9% of Se were found. The Se peak overlaps with the As peak causing small deviations in the Se content. Taking this into account, the ratio of Bi to Se from these measurements indicates the correct approximate stoichiometry for Bi_2_Se_3_.

**Figure 3:**
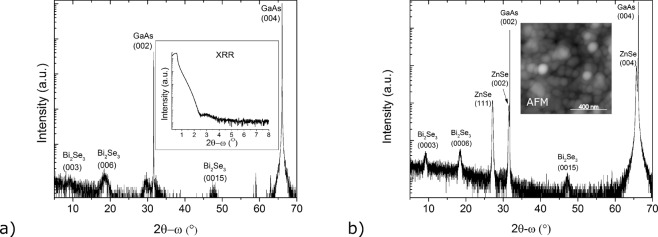
(**a**) High-resolution X-Ray diffraction of Bi_2_Se_3_ QDs. X-ray reflection (XRR) is shown in the inset. (**b**) High-resolution X-Ray diffraction pattern of Bi_2_Se_3_ QDs capped with 100 nm of ZnSe. The inset shows 1 µm × 1 µm AFM image of the surface.

High-resolution X-ray diffraction (HR-XRD) and X-ray reflection (XRR) experiments were also performed on the QDs. The results, after the alignment in the substrate (004) peak, are presented in Fig. 3a. The most intense Bi_2_Se_3_ peaks: (0003), (0006) and (00015) can be identified in the 2θ-ω scan. These demonstrate the presence of crystalline Bi_2_Se_3_ with the (0001) plane aligned to the (001) plane of the substrate. The noisiness and broadness of the peaks are not surprising due to the small amount of Bi_2_Se_3_ present on the surface and the inherent roughness of the QD layer. The roughness is also confirmed by the rapid damping observed in the measured XRR curve (Fig. 3a inset).

Since the GaAs substrate surface remains exposed, any epitaxial material compatible with GaAs could be grown by MBE to cap the QDs. The capping layer will primarily protect the Bi_2_Se_3_ QDs from exfoliation and oxidation, and it could also serve as a barrier for carrier confinement. Unfortunately, Bi_2_Se_3_ will desorb from the surface when the sample temperature is raised above 300 °C. Thus, further growth of GaAs could not be used for capping purposes. Zinc Selenide, ZnSe, a wide-gap semiconductor (E_g_ = 2.7 eV), has a very similar lattice parameter as GaAs and it can be grown as a strained layer on GaAs^34^. ZnSe has also been previously grown over Bi_2_Se_3_ layers^35^. The ideal temperature for ZnSe epitaxial growth is close to 250 °C, which is the temperature used for the QD growth.

Based on these considerations, another sample was grown where the QDs were capped with 100 nm of ZnSe. This growth took place in the same chamber, immediately after the QD growth. The zinc was provided by a RIBER Zn Knudsen effusion cell while the Se flux from the VCOR cell was maintained. The growth rate of ZnSe was 200 nm/h. The sample was exposed to Zn and Se fluxes for a period of time such that the overgrown layer thickness, assuming flat-layer growth, was ~100 nm. The HR-XRD pattern for the overgrown sample is shown in Fig. 3b. The Bi_2_Se_3_ peaks are still present as before, and narrow peaks corresponding to the (002) and (004) reflections of ZnSe can be observed adjacent to the corresponding GaAs peaks. The lattice mismatch relative to the GaAs is −0.57%, which means that the ZnSe layer should grow epitaxially and pseudomorphic to the GaAs substrate. From the AFM analysis, which is shown in the inset of Fig. 3b, it is notable that the surface is still rough even after growth of the capping layer (RMS smoothness ~7.4 nm) due to the initial roughness originating from the QDs, as well as ZnSe grains and defects. Furthermore, another peak is also visible in the HR-XRD pattern. This peak is attributed to the growth of grains of wurtzite ZnSe or of misoriented ZnSe on top of the Bi_2_Se_3_ QDs by van der Waals epitaxy^36^. Chen^36^ and Hernandez-Mainet^35^ *et al*., described the preferential growth of the wurtzite (hexagonal) phase when ZnCdSe was grown on Bi_2_Se_3_. However, we were unable to find the same described peaks or symmetry in the HR-XRD pattern. Instead, the peaks and symmetry observed indicate the presence of (111)-oriented ZnSe grains.

**Figure 4:**
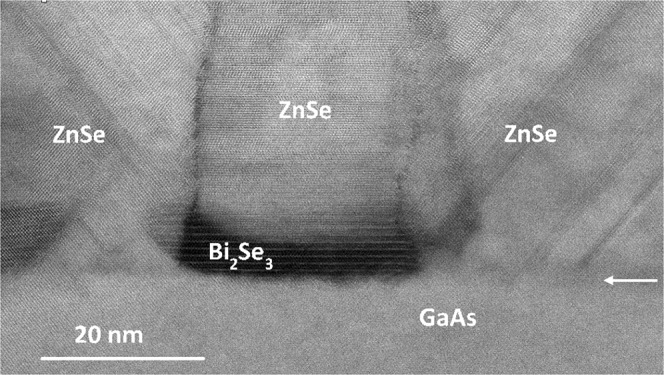
High-resolution scanning transmission electron micrograph recorded using large-angle bright-field imaging mode, showing Bi_2_Se_3_ QDs capped with 100 nm of ZnSe. Arrow indicates top of GaAs substrate.

**Figure 5:**
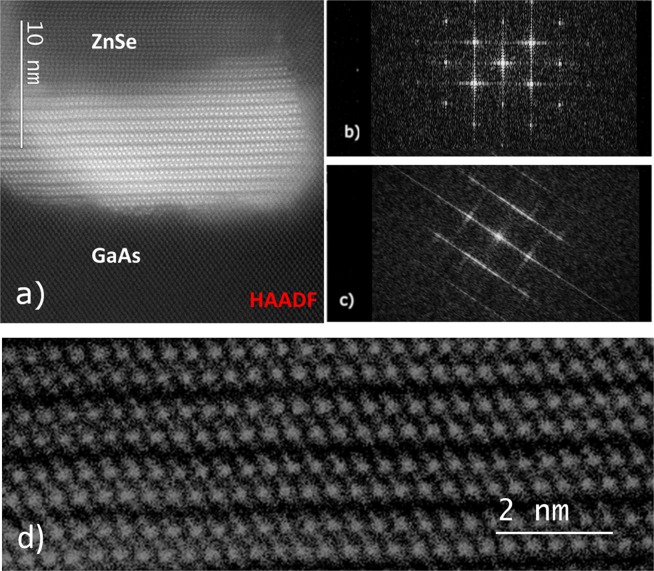
Aberration-corrected electron micrograph of Bi_2_Se_3_ QDs capped with 100 nm of ZnSe: (**a**) High-angle annular-dark-field (HAADF) image. (**b**) FFT of GaAs region in (**a**). (**c**) FFT of ZnSe region in (**a**) rotated by 45°. (**d**) Detail of quintuple-layer stacking visible inside the Bi_2_Se_3_ QD. Inverted contrast large-angle bright-field (LABF) image.

Aberration-corrected electron microscopy was used to image cross sections of the ZnSe-capped Bi_2_Se_3_ QDs: examples are shown in Figs 4 and 5. It is clear from Fig. 4 that the interface between GaAs and ZnSe is abrupt and continuous with few defects. Bismuth atomic columns appear with high contrast in both high-angle annular-dark-field (HAADF) and large-angle bright-field (LABF) image modes. Thus, the layered crystal (quintuple layer) structure of the Bi_2_Se_3_ QDs is readily identified in Fig. 4. Moreover, one can also clearly see the columnar growth of misoriented ZnSe, with stacking faults in the capping layer originating from the QDs or grain boundaries. Figure 5a,d clearly show the Bi_2_Se3 layered structure at very high resolution. The atomic stacking visible in the ZnSe capping layer directly above the Bi_2_Se_3_ QD in Fig. 5a suggests that ZnSe in this region grows as a disordered mixture of (0001) wurtzite and (111) zincblende structures, as previously observed in ZnSe nanobelt growth^37^. Fast Fourier Transforms (FFTs) of the TEM micrograph from areas above the QDs (Fig. 5c), on the sides of the QDs (i.e., on bare GaAs) (Fig. 5b), are very similar, despite the rotation, supporting the presence of tilted and defective zincblende ZnSe rather than the disordered wurtzite phase that is visible above the QDs. Finally, it is interesting that this image also suggests the presence of some bismuth at the grain boundaries, and the QD height is slightly greater close to the QD edge than in the QD center. This implies the occurrence of some Bi segregation during the capping process, similar to what has been reported for Indium in the InAs SK QDs^38^.

**Figure 6:**
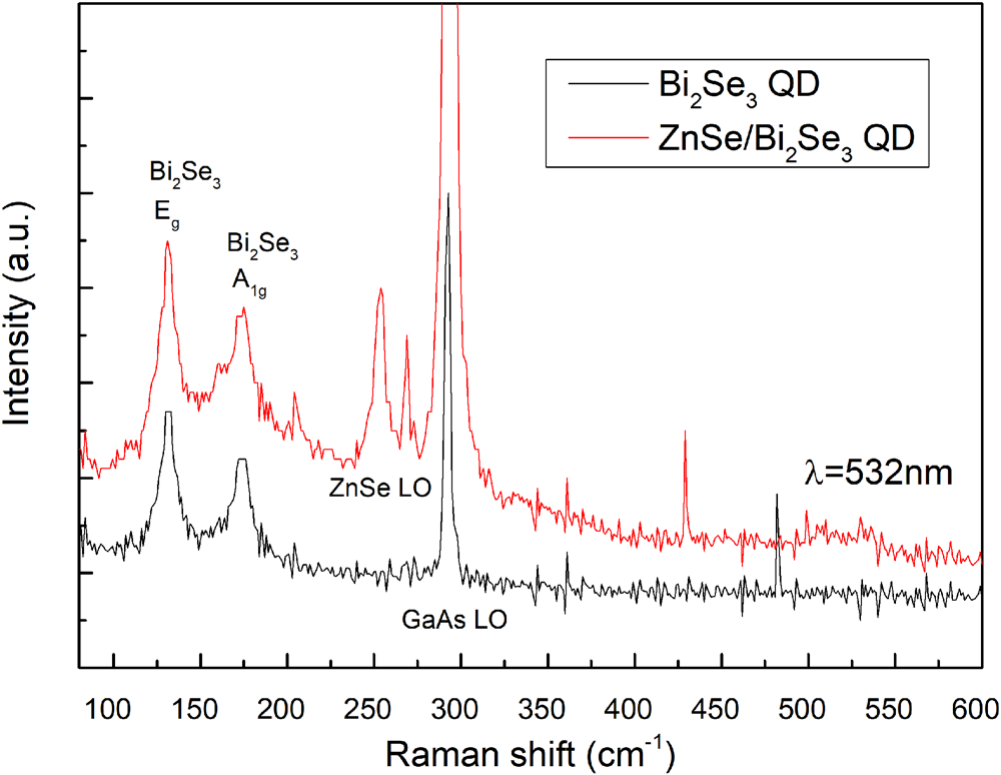
Room temperature Raman spectra of Bi_2_Se_3_ QDs uncapped and capped with ZnSe.

The Raman spectra of both samples is presented in Fig. 6. In the QD sample, the surface is mostly transparent and the GaAs LO (292 cm^−1^) substrate peak is the most intense, which is not the case in continuous films since the surface is reflective in the visible range due to the metallic behavior of the film. Two of the main peaks of bulk Bi_2_Se_3_ can be noted at 174 and 131 cm^−1^, and they correspond to the A_1g_ and E_g_ modes, respectively. These peaks are very similar in the capped and uncapped samples, and the only difference is the addition of the ZnSe LO peak (254 cm^−1^) from the cap layer. The Raman spectra shown in Fig. 6 confirm the high quality of the Bi_2_Se_3_ QDs. The positions of the E_g_ and A_1g_ modes do not present the blue-shift or the change in the intensity ratio that has been observed when the thickness is reduced to a few quintuple layers, and Bi_2_Se_3_ loses the TI properties (less than 6 quintuple layers)^39^ consistent with the observed QD thickness.

## Conclusion

The droplet epitaxy technique has been successfully applied to the growth of quantum dots of Bi_2_Se_3_, which is a layered van der Waals material that is not amenable to the more conventional strain-driven Stranski-Krastanov techniques of self-assembly via MBE. The Bi_2_Se_3_ QDs can be grown with high density and they have good crystal quality and electronic properties. Both uncapped and ZnSe-capped Bi_2_Se_3_ QDs were demonstrated. The size achieved is opportune, since electron confinement for a 3D Topological insulator would be anticipated. The QD formation process, as with other self-assembled techniques, is simple, reproducible and tunable, and should be ideal for the fabrication of large-area devices and experiments wherein the signal of several comparable quantum dots is required. The results offer the means to explore new physics and device applications possible with these unique low dimensional structures.

## Methods

The HR-XRD measurements were performed using a Bruker D8 Discover 3D diffractometer with a da Vinci configuration, 1-mm Slit/Collimator, and a Cu Kα1(1.5418 Å) source. The AFM images were captured with a Bruker Dimension FastScan AFM with a FastScan-A silicon probe and the SEM images with a FEI Helios Nanolab 660. The aberration-corrected imaging was carried out with a JEOL ARM-200F scanning transmission electron microscope operated at 200 keV. The probe convergence angle was set at 20 mrad, and the image collection angles were 0–22 mrad for large-angle bright-field imaging and 90–150 mrad for high-angle annular-dark-field imaging. The Raman spectra were performed on a WiTec confocal microscope using a 1 mW 532 nm laser focused by a 50x lens.

## Data Availability

The datasets generated during and/or analyzed during the current study are available from the corresponding author on reasonable request.
